# Influence of the COVID-19 Lockdown on the Physical and Psychosocial Well-being and Work Productivity of Remote Workers: Cross-sectional Correlational Study

**DOI:** 10.2196/30708

**Published:** 2021-12-01

**Authors:** Yessica Abigail Tronco Hernández, Fabio Parente, Mark A Faghy, Clare M P Roscoe, Frances A Maratos

**Affiliations:** 1 School of Health Professions University of Plymouth Plymouth United Kingdom; 2 School of Psychology College of Health, Psychology and Social Care University of Derby Derby United Kingdom; 3 School of Human Sciences College of Science and Engineering University of Derby Derby United Kingdom

**Keywords:** COVID-19, pandemic, remote workers, mental health, health policy, employment, policymakers, wellbeing

## Abstract

**Background:**

Lockdowns imposed during the COVID-19 pandemic have impacted the living and working habits of millions of people, with potentially important implications for their physical, mental, and social well-being.

**Objective:**

The primary objective of this study was to investigate the impact of the COVID-19 pandemic on remote workers who were not directly affected by COVID-19.

**Methods:**

This was a correlational cross-sectional study (with an additional qualitative component) of 184 remote workers surveyed during the first COVID-19 lockdown in the United Kingdom. Standard measures of mental health (Kessler-6 Distress Scale), productivity (Brief Instrument to Assess Workers’ Productivity During a Working Day), and physical activity (International Physical Activity Questionnaire) were used, and respondents were further surveyed on changes to their dietary, exercise, smoking, drinking, and socialization habits to produce a well-being change index.

**Results:**

The results revealed associations between sedentary behavior and poorer mental health (*τ*_b_=0.14) and between poorer mental health and low work productivity (*τ*_b_=–0.39). However, both positive and negative lifestyle changes were reported; a self-reported increase in well-being (with respect to diet, exercise, smoking, alcohol consumption, and socialization) since the start of the pandemic was associated with both better mental health (*τ*_b_=–0.14) and better work productivity (*τ*_b_=0.14). Of note, among respondents without a mental health diagnosis (137/184, 74.4%), we observed rates of moderate (76/137, 55.5%) and severe (17/137, 12.4%) psychological distress, which were markedly higher than those reported in large prepandemic studies; moreover, 70.1% (129/184) of our respondents reported more sedentary behavior, 41% (69/168) increased their alcohol consumption, and 38.6% (71/184) increased their overall food intake. However, 46% (75/163), 44.8% (39/87) and 51.8% (57/110) of respondents reported spending more time walking and engaging in more moderate and vigorous exercise, respectively. Qualitative analysis revealed many positive adaptations to lockdowns (eg, decreased commuting expenses, flexibility) but also a number of structural obstacles to remote working (eg, lack of support and high expectations from employers, childcare duties).

**Conclusions:**

These findings may be of practical importance for policy makers and employers in a world in which work involves long-term remote or hybrid employment arrangements; strategies to promote more sustainable remote working are discussed.

## Introduction

### Background

The COVID-19 pandemic has had catastrophic effects on global economies, with significant reductions in commercial and business activities projected [1] as well as increasing unemployment and underemployment with associated loss of income [2,3]. In a study of Vietnamese remote workers, 61% of respondents reported losses of income as a result of the country’s first national lockdown, with women more financially affected than men [4]. The COVID-19 pandemic has further forced a work strategy paradigm shift in a very short period of time, and it does not provide the flexibility that home working would offer under normal conditions [5]. In several industries, working remotely has become a prominent solution to continued employment (eg, higher education delivery; business and operational management; administrative/secretarial work) during the pandemic. With uncertainty surrounding the prolonged impacts of COVID-19, and companies accelerating their plans to shift to remote working as a new default [6,7], there is an urgent need to understand the direct and indirect impact of remote working [8]. The impact of such sudden changes to working routines needs to be addressed in an attempt to understand the broad impacts of COVID-19 on work productivity and well-being [9].

In the United Kingdom, lockdown and social distancing measures were imposed starting in March 2020 [10]. By April, almost half of UK employees were working remotely, 90% of them having transitioned to this form of working because of the lockdown [10]. However, to date, little attention has been directed toward understanding the health, well-being, and societal impacts of remote working. This has most likely reflected (1) the need to increase epidemiological understanding and direct impacts on frontline services and patients; (2) prepandemic evidence demonstrating the benefits of remote working due to its flexibility [11] and financial convenience [12]; and (3) the low risk that people working remotely will contract the infection due to reduced social contact and isolation [13]. However, the validity of prepandemic evidence is questionable in the current climate, where societal and economic issues are profoundly different. Accordingly, this paper will present insight into the effects of remote working to establish an understanding of its impacts upon physical health, psychosocial well-being, and work productivity.

Although remote (or distant) working is not a new phenomenon, before 2000, only 2.5% of UK workers (2/3 of them women) worked remotely. Historically, the logic behind flexible work arrangements has been to avoid losing valuable labor to factors such as childcare and family commitments [5,14], as well as to promote a more environmentally friendly way of working (eg, decreasing resources to commute) [15,16]. Well-being has been identified as a key factor behind productive remote working [17,18]. However, as a consequence of COVID-19, the number of people working remotely in the United Kingdom has increased to 13.02 million [10]. Thus, there is an urgent need to understand the ramifications of this unprecedented switch in employment type, including resultant well-being and productivity lifestyle changes. Although well-being is a complex and multifactorial state, key facets include diet, exercise (physical health), and mental health [19,20], which are each linked to societal, economic, and mortality issues.

### Well-being and Work Productivity

Mental health disorders account for a significant proportion of the global disease burden; together with worker burnout, it is estimated that they are currently costing the global economy over US $1 trillion per year and will cost $16 trillion per year by 2030 [21]. Reports have already been published of physical and emotional burnout, as well as mental health difficulties, among physicians and nurses [22-25] as well as among social carers [26,27], pointing to a clear link between mental health and work productivity [28]. Holmes and colleagues [29] report that major adverse consequences of the COVID-19 pandemic are likely to be social isolation and loneliness; both of these can lead to depression, anxiety, self-harm, and attempted suicide. Social isolation and loneliness are factors that can exacerbated by remote working, especially where the switch has been rapid and unexpected. Indeed, Holmes et al [29] further suggest that working from home, as a consequence of the pandemic, has abruptly interrupted many social opportunities that are important for physical and psychological health.

Remote working may also allow for greater media consumption, which in turn has been correlated with anxiety and depression amid the current pandemic [30]. Moreover, following the Ebola crisis, media exposure was found to exacerbate stress responses and worries [31], and messaging regarding Ebola risks was found to increase public anxiety [32]. Thus, increased consumption of media during times of crises and pandemics may be a maladaptive coping consequence. For example, Jungmann and Witthöft [33] have reported that both health anxiety and cyberchondria (excessive searching for information on the web) constitute risk factors for COVID-19 anxiety. However, they further observed that adaptive emotion regulation (in this particular case, using cognitive emotion regulation strategies to cope with negative life events) protected against COVID-19 anxiety. Consistent with this, in a sample of over 5000 Spanish adults surveyed during the Spanish lockdown, Fullana and colleagues [34] found that consuming a healthy diet and avoiding high consumption of COVID-19 news predicted lower reports/symptoms of depression and anxiety, as did taking the opportunity to pursue hobbies and engaging with nature (even if just looking outside).

During the COVID-19 pandemic, people’s eating habits have been shown to be unhealthier, particularly those relating to snacks and alcohol consumption [35]. Obesity and its related comorbidities are additionally cited as major risk factors for COVID-19 infection [36,37] and poorer clinical outcomes [38]. Of direct relevance is the recently launched “Better Health” campaign by the UK government, which aims to support actions against COVID-19 and reduce obesity-related costs in the National Health Service (£6 billion [US $4,334,260] per year [39]). Hence, diet has a crucial role in preserving health and protecting at-risk populations during the COVID-19 pandemic. As such, it is essential to understand how diet has changed as a consequence of COVID-19 work pattern changes, including the potential added factor of sedation (ie, physical inactivity and increased sedentary behaviors).

The World Health Organization (WHO) has classified physical inactivity (6%) as the fourth leading risk factor of global mortality, after hypertension (13%), smoking (9%), and diabetes (6%) [40]. The WHO recommends 60 minutes per day of moderate to vigorous physical activity for youth aged 6-17 years and 75-150 minutes per week of vigorous or moderate physical activity for adults and older persons, respectively, including 3 and 2 days per week each of muscle- and bone-strengthening activities (eg, resistance training) [41]. COVID-19 has had a major impact on physical activity behaviors, due to movement (even leaving one’s residence) and self-isolation restrictions for prolonged periods [42]. Ammar and colleagues [35] report that COVID-19 home confinement has negatively affected all physical activity intensities (light, moderate, vigorous, and overall), while sedentary behaviors such as sitting, lying down, or screen use (eg, TV viewing, video game playing) have increased from 5 to 8 hours per day, despite widespread access to web-based physical activity training programs or workouts [43].

Prior to the COVID-19 pandemic, physical inactivity was costly and was recognized as the fourth leading cause of mortality by the WHO [44]. For example, in 2013, it was reported that physical inactivity cost health care systems worldwide US $53.8 billion [45], with deaths attributable to physical inactivity costing a further $13.7 billion in productivity losses [46]. Sedentary behaviors (independent of physical inactivity) are further associated with cardiovascular risk factors and increased cardiovascular morbidity and global mortality [47]. Unfortunately, since the start of the COVID-19 crisis, restrictions have removed many opportunities to be physically active and reduce sedentary behaviors. The global ramifications of this are concerning, as individuals who were not active before COVID-19 are now at even more risk of cardiometabolic abnormalities, sarcopenia, and frailty in older persons [48]. This scenario has been referred to as “two pandemics”—one pandemic being COVID-19, and the second consequential pandemic being physical inactivity [46].

In sum, an individual’s ability to maintain a healthy diet, physical activity, and good mental health have likely been impacted by transitioning to remote working. The pandemic has further added several obstacles to the world of work (eg, childcare duties given school closures, which could disproportionately affect women) [49]. Consequently, there is an urgent need to better understand how, for those in employment, the abrupt switch to remote working (and, more generally, remote working during a pandemic) has affected mental and physical health, including general patterns of change in well-being. These findings will also inform our understanding of the public health implications of a long-term or permanent shift to remote working or hybrid arrangements for many people, even after the end of the pandemic. As such, our goals were to explore (1) the relationship between physical activity, mental health, diet, and work productivity during the initial COVID-19 lockdown period; (2) the demographic characteristics associated with varying well-being in this population; and (3) the perceptions remote workers had of their well-being and its influence on work productivity.

## Methods

### Design

A correlational design was employed to investigate associations between standard indices of mental health, physical activity, and productivity and ad hoc measures of changes in physical activity, dietary habits, and smoking habits. Open-ended questions were also posed to further probe diet, and a final question asked remote workers about the perceptions had of their well-being in relation to work productivity.

### Respondents

Following ethical approval by the local university, the survey was circulated to adult residents of the United Kingdom on social media (ie, Facebook and Twitter) and through press releases between May 15 and July 6, 2020. The latter date marked the beginning of the first week during which a number of indoor amenities (eg, museums, places of worship, libraries) and hospitality facilities (cafes, pubs, and restaurants) reopened in England [50].

Between these dates, data were collected from 279 respondents, of whom 207 were remote workers at the time. Of these, 25 respondents did not complete all compulsory aspects and were therefore excluded. This left a final sample of N=184, of whom 167 (90.7%) were not remote workers before the lockdown (ie, before March 23, 2020 in the United Kingdom). Based on power analysis for a correlational design, assuming *r*=0.3 and with *α*=.005, we estimated that N=142 should be sufficient to have 0.8 power to detect such relationships.

### Measures

The survey included quantitative standardized measures of mental health, physical activity, and work productivity as well as an open qualitative question asking respondents to provide any additional information about their lockdown experiences that was not covered by the questionnaire measures and further quantitative items. These further quantitative items were used to probe dietary habits, socialization, and activities used as coping mechanisms to preserve well-being during the lockdown (see specifically *Measures of Diet and Well-being Change During the Lockdown* and *Socialization, News Consumption, and Coping Strategies*).

### Kessler-6 Distress Scale

The Kessler-6 Distress Scale (K6) [51] was administered as a measure of psychological distress. The K6 asks respondents to rate the degree to which, in the past 30 days, they have experienced nervousness, hopelessness, restlessness, depression, and feelings of worthlessness on a Likert scale with responses ranging from 1, all of the time, to 5, none of the time. The scale produces a potential score range between 0 and 24, with scores ≥5 generally considered markers of moderate distress and scores of ≥13 considered markers of high psychological distress and serious mental illness [52]. The scale has good internal consistency [51], *α*=.89.

### International Physical Activity Questionnaire

The short version of the International Physical Activity Questionnaire (IPAQ) for middle-aged adults [53,54] was used to measure the degree of physical activity or sedentarism. The questionnaire asks respondents to estimate (1) the number of days they spent more than 10 minutes walking or engaging in moderate (eg, cycling, doubles tennis) and vigorous (eg, heavy lifting, fast cycling) exercise over the past 7 days; (2) the number of minutes they spent walking or engaging in these activities during the average day over this period; and (3) the number of hours they spent sitting per average day. Physical activity is categorized by intensity and includes sedentary behaviors, as well as light, moderate, and vigorous physical activity levels. Metabolic equivalents (METs) are then commonly used to express the intensity of the physical activities reported. A MET is defined as the ratio of an individual’s working metabolic rate to their resting metabolic rate. A MET equates with the oxygen consumption required at rest/sitting quietly and is assumed to be 3.5 mL/O_2_/min × kg body weight [55]. In sedentary behavior (as defined above), the energy expenditure is less than 1.5 METs [56]. It is suggested that compared with sitting quietly, a person's caloric consumption is 3 to 6 times higher when they are moderately active (3-6 METs) and more than 6 times higher when vigorously active (>6 METs). The scale has acceptable internal consistency [57], *α*=.60.

### Brief Instrument to Assess Workers’ Productivity During a Working Day Scale

Work productivity was assessed using the Brief Instrument to Assess Workers’ Productivity During a Working Day (IAPT) [58]. This 10-item instrument asks respondents to rate the degree to which they have felt focused, tired/sleepy, confident, productive, annoyed/upset, satisfied, or affected by physical symptoms such as pain or dizziness over the last two hours of work. Ratings are given on a scale of “not at all” to “extremely,” which is scored between 0 and 4. This produces an overall score ranging from 0 to 40 points, with higher scores denoting higher productivity. The scale has good split-half reliability (*r*^2^=0.86), good internal consistency (*α*=.80-.91), and high convergent validity (*r*^2^=0.86) with longer instruments such as the Health and Work Performance Questionnaire [59].

### Measures of Diet and General Well-being Change During the Lockdown

A total of 9 items were used to assess whether respondents had experienced an increase, decrease, or no change (3 response options) in overall food consumption, which included consumption of fruits, vegetables, snacks, treats, takeaway food, home cooking, soft drinks, and alcoholic drinks. Similarly, 4 items probed whether the time individuals had spent walking, sitting, or engaging in moderate and vigorous physical activity had changed since the lockdown. Respondents were also asked whether they had started or quit smoking since the start of the lockdown, and whether the amount they smoked had increased, decreased, or stayed the same. Lastly, respondents were asked whether the amount they socialized (including virtually) with others had increased, decreased, or stayed the same since the lockdown.

Measures of diet and well-being change during the lockdown were coded as 0 for no change and +1 or 1 for a decrease or increase depending on the item, respectively. The full coding scheme is presented in Table 1. Responses were then aggregated into a well-being change index (WCI) since the start of the lockdown, with scores ranging from –16 to +16, with higher values typically indicating improved overall general well-being.

**Table 1:** Scoring scheme for the questionnaire items directly probing habit changes since the start of the lockdown.

Measure	More than before	No change	Less than before
Overall food intake	–1	0	1
Snacks	–1	0	1
Treats	–1	0	1
Sugar/fizzy drinks	–1	0	1
Alcohol	–1	0	1
Take-away food	–1	0	1
Sitting	–1	0	1
Smoking^a^	–1	0	1
Smoking frequency	–1	0	1
Fruits	1	0	–1
Vegetables	1	0	–1
Cooking/baking	1	0	–1
Walking	1	0	–1
Moderate exercise	1	0	–1
Vigorous exercise	1	0	–1
Socializing	1	0	–1

^a^Smoking initiation (“more than before”) or cessation (“less than before”) since the start of the lockdown.

### Socialization, News Consumption, and Coping Strategies

Respondents were also asked to estimate the average amount of time (in minutes per day) that they spent socializing with individuals within and outside their household, and the amount of time (in minutes per day) they spent consuming news content (in print, on the internet, or on TV/radio). Respondents were further asked to select all the resources and strategies they had engaged in to maintain their physical and mental well-being during the lockdown.

The list of resources and strategies for physical activity included already-owned implements, newly purchased implements, specialized books and magazines, smartphone apps, web pages, TV programs, and advice from friends and family. This yielded a possible range of counts between 0 and 7.

The list of resources and strategies for mental well-being included yoga, meditation, prayer and other spiritual practices, counseling, reading, watching TV, playing video games, and keeping a diary. Respondents were also given the opportunity to list any further mental well-being coping strategies they were employing. These were counted and added to the overall count. This yielded a range of responses between 0 and 13.

### Open-Question Self-reports

Respondents were given the opportunity to enter text (3000 characters maximum) to volunteer additional information on any of the aspects probed by the survey (diet, mental health, exercise, and work productivity) or to mention anything not covered by the survey that they felt was relevant to their experiences of well-being changes during the COVID-19 lockdown.

### Demographics

Lastly, respondents were asked several demographic (age group, gender, educational attainment) and household questions (marital status, whether they had adult or underaged children, whether they lived with other adults).

### Procedure

Following informed consent, respondents selected a 6-digit alphanumeric code used to anonymize their data and allow for retrieval. They were then presented with items/questionnaires regarding, in order, work productivity (IAPT), dietary changes, mental health (K6), physical activity (IPAQ), exercise resources, and coping strategies. These were followed by the optional open-ended question and, finally, the demographics questions.

### Data Analysis

Kolmogorov-Smirnov tests of normality were conducted on key measures (IAPT, K6, WCI, METs, and sitting time); all significantly deviated from normality (*P*<.05). Visual inspection of the correlation plots for these measures additionally revealed substantial nonlinearity in the relationships between several of them. For this reason, Kendall *τ*_b_ correlations were performed to detect statistically significant relationships between psychophysical well-being and productivity. Independent-sample Mann-Whitney *U* tests and chi-square analyses were used to test for differences in exercise habits, mental health scores, and productivity between demographics (focusing on gender differences and childcare responsibilities). Missing cases were excluded pairwise to maximize the amount of data available for analysis.

The open-ended question responses were analyzed using conventional content analysis [60], conducted by YATH and following the eight steps suggested by Zhang and Wildemuth [61], which involved preparing data, coding texts, and making inferences from the meanings of the data. This allowed for the observation of trends in the respondents’ opinions. To increase the trustworthiness of the data, triangulation was conducted with the quantitative results, reflexivity was included across data collection and analysis, and peer debriefing was conducted with other members of the research team [62].

### Ethical Considerations

The study was approved by the University of Derby College of Life and Natural Sciences Research Ethics Committee (ETH1920-3136). Participants provided informed consent at the start of the web-based survey.

## Results

### Relationships Between Physical Activity, Dietary and Well-being Changes, Mental Health, and Productivity

**Figure 1:**
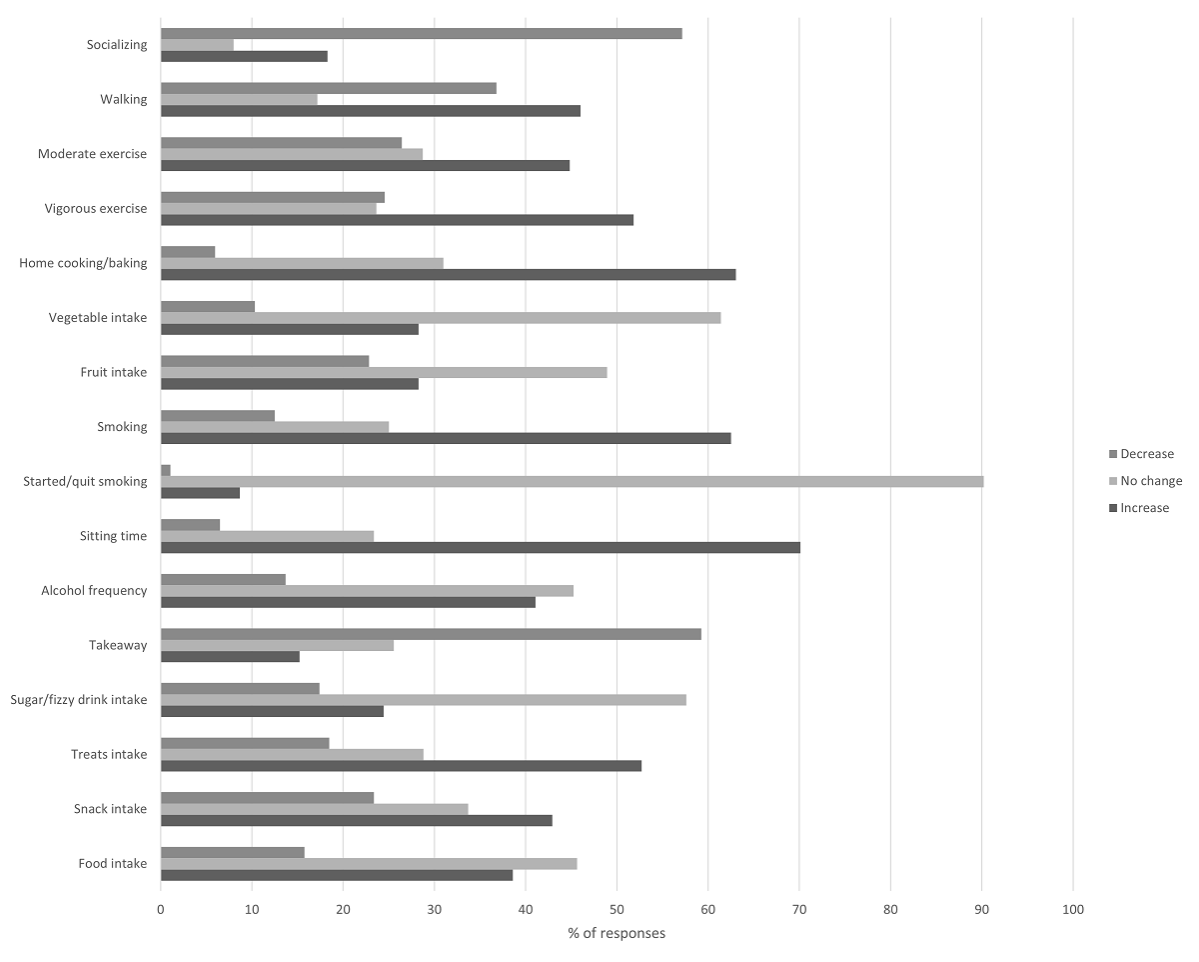
Respondents’ reported changes in dietary habits, physical activity, socialization, and general well-being as a function of lockdown during the COVID-19 pandemic (metrics were computed by following the scheme in Table 1). Bars indicate the percentages of respondents reporting a decrease (top bar in each cluster), increase (bottom bar), or no change (middle bar).

Descriptive statistics for standardized measures of productivity (IAPT), mental health (K6), physical activity (IPAQ, expressed in METs), time spent sitting, and well-being change since the lockdown (WCI) are presented in Table 2. Figure 1 shows the distribution of responses for the WCI components.

An initial round of one-tailed correlations (with the *α* level set at *P*<.005) was computed between respondents’ productivity scores (IAPT), mental health scores (K6), aggregated well-being change scores (WCI), MET measures derived from the IPAQ, and reported time spent sitting. The results are reported in Table 3 (sections 1-8) and suggest relationships between sedentarism, poorer mental health, a decrease in well-being, and productivity. Namely, the more time respondents reported spending sitting, the worse their mental health scores (K6) and the lower their productivity (IAPT); similarly, a decrease in reported well-being since the start of the lockdown (WCI) was associated with worse productivity and poorer mental health.

Given the observed relationship between physical activity, mental well-being, and productivity, we tested for differences in the above measures between individuals with and without a reported pre-existing mental health diagnosis (45/184, 24.4%, and n=137/184, 74.5%, respectively, as 2 respondents did not provide this information). The results of the independent-sample Mann-Whitney *U* test are reported in Table 4. As expected, respondents with a previous mental health diagnosis reported significantly worse mental health, engaged in significantly less vigorous exercise, and spent more time sitting than those without a pre-existing diagnosis.

Excluding the subset of respondents (45/184) with a pre-existing mental health diagnosis (80% [36/45] of whom had K6 scores ≥5 and 22.2% [10/45] of whom had K6 scores ≥13), 55.5% (76/137) of the remaining respondents had scores consistent with moderate distress and 12.4% (17/137) had scores consistent with severe distress. For context, a survey of over 50,000 noninstitutionalized Californian adults under nonpandemic conditions [52] yielded incidences of 27.9% with scores ≥5 and 8.6% with scores ≥13.

**Table 2:** Descriptive statistics for the assessed measures of physical activity (IPAQ METs), sitting time (hours per average day), mental health (K6), work productivity (IAPT), and well-being change (WCI).

Measure	Range (IQR)	Mean (SE)	95% CI
IAPT^a^	1 to 39 (11)	21.61 (0.511)	20.60 to 22.62
K6^b^	0 to 24 (7)	6.94 (0.361)	6.23 to 7.65
WCI^c^	–10 to 12 (7)	–0.28 (0.310)	–0.89 to 0.33
Vigorous METs^d^	0 to 5040 (1440)	827.17 (75.95)	677.31 to 977.04
Moderate METs	0 to 3840 (360)	286.80 (39.90)	208.08 to 365.53
Walking METs	0 to 3465 (610.50)	645.87 (44.19)	558.68 to 733.06
Total METs	0 to 6993 (2033.88)	1759.85 (104.41)	1553.83 to 1965.86
Sitting time	2 to 18.0 (3.0)	8.81 (0.238)	8.34 to 9.28

^a^IAPT: Brief Instrument to Assess Workers’ Productivity During a Working Day.

^b^K6: Kessler-6 Distress Scale.

^c^WCI: well-being change index.

^d^METs: metabolic equivalents.

**Table 3:** Results of both rounds of Kendall *τ*_b_ correlations between productivity (IAPT), mental health (K6), IAPT METs, sitting time, resources for physical activity, and general coping activities. News intake (minutes per average day) and total time spent socializing (within and outside the household) did not produce any significant correlations (at *P*<.005) and were therefore excluded from the table. Italic text indicates significance at *α*=.005.

Measure		IAPT^a^	K6^b^	WCI^c^	Vigorous METs^d^	Moderate METs	Walking METs	Total METs	Sitting time	Exercise resources	Coping activities
**IAPT**											
	*τ*_b_	1	–*0.393*^e^	*0.178* ^e^	0.038	0.073	0.108	0.090	–0.107	0.010	–0.074
	*P* value	—	*<.001*	*<.001*	*.24*	.10	.02	.04	.02	.43	.09
**K6**											
	*τ*_b_	–*0.393*^e^	1	–*0.148*^f^	–0.081	–0.108	–0.102	–0.121	*0.147* ^f^	0.050	0.089
	*P* value	*<.001*	—	.003	.07	.03	.03	.009	*.003*	.19	.054
**WCI**											
	*τ*_b_	*0.178* ^e^	–*0.148*^f^	1	0.143	0.001	0.105	0.133	–0.097	0.112	0.028
	*P* value	*<.001*	*.003*	—	.005	.50	.02	.005	.04	.03	.31
**Vigorous METs**											
	*τ*_b_	0.038	–0.081	0.143	1	*0.224* ^e^	0.057	*0.646* ^e^	–*0.256*^e^	*0.219* ^e^	0.036
	*P* value	.24	.07	.005	—	*<.001*	*.14*	*<.001*	*<.001*	*<.001*	*.*26
**Moderate METs**											
	*τ*_b_	0.07	–0.108	0.001	*0.224* ^e^	1	0.027	*0.379* ^e^	–*0.180*^f^	0.157	0.060
	*P* value	.10	.03	.50	*<.001*	—	*.31*	*<.001*	*.001*	.005	.15
**Walking METs**											
	*τ*_b_	0.108	–0.102	0.001	0.105	0.027	1	*0.361* ^e^	–0.134	–0.018	–.007
	*P* value	.019	.025	.496	.023	.312	—	*<.001*	.006	.378	.448
**Total METs**											
	*τ*_b_	0.090	–0.121	0.133	*0.646* ^e^	*0.379* ^e^	*0.361* ^e^	1	–*0.269*^e^	*0.151* ^f^	0.008
	*P* value	.04	.009	.005	*<.001*	*<.001*	*<.001*	—	*<.001*	*.003*	.44
**Sitting time**											
	*τ*_b_	–0.107	*0.147* ^f^	–0.097	–*0.256*^e^	–*0.180*^f^	–0.134	–*0.269*^e^	1	–0.139	–0.070
	*P* value	.02	*.003*	.04	*<.001*	*.001*	.006	*<.001*	—	.008	.11
**Exercise resources**											
	*τ*_b_	0.010	0.050	0.112	*0.219* ^e^	0.157	–0.018	*0.151* ^f^	–0.139	1	*0.240* ^e^
	*P* value	.43	.19	.03	*<.001*	.005	.38	*.003*	.008	—	*<.001*
**Coping activities**											
	*τ*_b_	–0.074	0.089	0.028	0.036	0.060	–0.007	0.008	–0.070	*0.240* ^e^	1
	*P* value	.09	.05	.31	.26	.15	.45	.44	.11	*<.001*	—

^a^IAPT: Brief Instrument to Assess Workers’ Productivity During a Working Day.

^b^K6: Kessler-6 Distress Scale.

^c^WCI: well-being change index.

^d^METs: metabolic equivalents.

^e^*P*<.001.

^f^*P*<.005.

**Table 4:** Physical activity (IPAQ METs and sitting time), mental well-being (K6), well-being change (WCI), and productivity (IAPT) measures compared between respondents with or without a mental health diagnosis.

Measure		Pre-existing diagnosis				*U*		*z*		*P* value
		Yes (mean rank)		No (mean rank)						
IAPT^a^		78.97		95.62		2518.50		–1.84		.07
K6^b^		116.10		83.42		1975.50		–3.62		<.001^c^
WCI^d^		84.03		93.95		2746.50		–1.10		.27
Vigorous METs^e^		71.27		98.15		2172.00		–3.07		.002^f^
Moderate METs		87.52		92.81		2903.50		–0.63		.53
Walking METs		95.60		90.15		2898.00		–0.60		.55
Total METs		82.99		94.30		2699.50		–1.24		.21
Sitting Time		111.49		84.22		2138.00		–3.05		.002^f^

^a^IAPT: Brief Instrument to Assess Workers’ Productivity During a Working Day.

^b^K6: Kessler-6 Distress Scale.

^c^*P*<.001.

^d^WCI: well-being change index.

^e^METs: metabolic equivalents.

^f^*P*<.005.

### Supplementary Analyses

#### Socialization and Coping Strategies

To gain a better understanding of how respondents were affected by lockdown social restrictions and how these are related to coping strategies, including resources individuals employed to maintain physical and mental well-being, we performed a second round of correlations (with the *α* level again set at *P*<.005). As such, correlations were computed for the respondents’ work productivity and mental health scores, physical activity (MET) scores, and reported sitting times (see Table 2 for descriptive statistics), together with the total number of physical activity resources (median 1, SD 1.15) and general coping activities (median 3, SD 1.78) that the respondents reported using or engaging in, the total amount of time they reported socializing with people within (mean 192.8 minutes, SE 15.4) and outside (mean 78.9 minutes, SE 6.15) their household, and their news intake (mean 50 minutes, SE 4.04). These results revealed no significant relationships between time spent socializing and any further measures.

Table 5 shows what part of our sample reported engaging in the different coping activities we provided. Respondents also had the option of mentioning activities not included on the list; some of the most frequently provided responses were arts, crafts, and general do-it-yourself activities (35/184, 19%), gardening (16/184, 8.7%), and cooking/baking (13/184, 7.1%).

**Table 5:** Respondents who reported engaging in different coping activities to maintain their psychophysical well-being (N=184).

Activity	Value, n (%)
Yoga	59 (32.1)
Meditation	30 (16.3)
Prayer/spiritual practices	12 (6.5)
Counselling/therapy	12 (6.5)
Reading	113 (61.4)
Watching TV	142 (77.1)
Playing video games	44 (23.9)
Keeping a diary	14 (7.6)
Other	62 (33.6)

#### Household and Gender Differences

Next, we aimed to investigate whether key demographic factors influenced respondents’ psychophysical and social well-being during the lockdown, as well as their coping strategies.

Here, we used the independent-sample Mann-Whitney *U* test to compare key measures between respondents from households with (n=46) and without (n=136) children under the age of 18 years. The results are reported in Table 6. Adults living in households with children reported, on average, approximately 2 hours less of sitting time and reported resorting to fewer recreational activities to maintain their psychosocial well-being. No other difference (eg, in mental health or productivity scores) achieved significance at the .005 *α* level.

Comparing the same measures as in Table 6 between men (n=40) and women (n=143) similarly revealed that women reported engaging in more recreational activities than men (mean ranks of 63.06 and 100.09, respectively) to maintain their psychosocial well-being (*U*=1702; *z*=–3.98; *P*<.001). Women were also significantly more likely than men to report being the main providers of childcare (*χ^2^_2_*=17.08; *φ*_c_=0.609; *P*<.001), and homeschooling (*χ^2^_2_*= 9.21; *φ*_c_=0.458; *P*=.01) in the household. No significant gender differences were found in the total number of physical activity resources that respondents reported using, *P*=.92.

**Table 6:** Physical activity, mental well-being, and productivity measures of respondents with and without children under 18 years of age.

Measure	Children aged <18 years in household, mean rank		*U*	*z*	*P* value
	Yes	No			
IAPT^a^	95.26	90.23	2955.00	–0.561	.57
K6^b^	84.48	93.88	2805.00	–1.04	.29
WCI^c^	92.13	91.29	3099.00	–0.094	.92
Socialization (in)	102.44	82.36	2042.50	–2.23	.02
Socialization (out)	88.48	90.51	2946.50	–0.23	.81
News intake	79.47	92.70	2471.00	–1.48	.13
Coping activities	64.64	100.58	1892.50	–4.07	<.001^d^
Vigorous METs^e^	80.79	95.12	2635.50	–1.65	.09
Moderate METs	87.61	92.82	2949.00	–0.63	.52
Walking METs	92.58	91.14	3078.50	–0.16	.87
Total METs	79.87	95.46	2589.00	–1.74	.08
Sitting time	64.10	100.17	1867.50	–4.06	<.001^d^

^a^IAPT: Brief Instrument to Assess Workers’ Productivity During a Working Day.

^b^K6: Kessler-6 Distress Scale.

^c^WCI: well-being change index.

^d^*P*<.001.

^e^METs: metabolic equivalents.

### Qualitative Self-report Data

Three themes emerged from the content data analysis related to different aspects of remote working. These were barriers to remote working and well-being preservation, mixed feelings and attitudes toward remote working, and aids to improve physical and psychosocial well-being. This section presents a narrative analysis of these themes with supporting illustrative respondent extracts.

#### Theme 1: Barriers to Remote Working and Well-being Preservation

This theme dealt with aspects of the lockdown that represented limitations to working and maintaining health and well-being at the standards individuals would have liked. Some respondents mentioned childcare responsibilities as a constraint, others mentioned how their eating habits had worsened, and some respondents reported difficulties in engaging with remote working.

Various respondents that were engaged in childcare duties described how stressful and tiring their work responsibilities were and how challenging it was to take care of themselves (health wise).

I have struggled to separate work and home learning with children

R9

…but have an 18-month-old also at home full time so productivity goes out the window; we have to organise our diaries at the start of the day so that we can pass her back and forth between us

R23

I have however been very unproductive work-wise, as my husband is still working full time and I have 2 young children to home school

R31

I have a child (3 years old) and having him off nursery […] dramatic impact on my mental health as I struggle to move from “mum mode” into “work mode” and has an impact about how I feel about my lack of work achievement – this then becomes a cycle of feeling as though I’m not achieving anything along with feeling mum guilt for not being with my son

R51

Respondents who reported having teaching and pastoral occupations recorded feeling more tired, stressed, and anxious. Moreover, there was an overall fear of losing their job and of not being “as productive” as expected if they could not adapt successfully to working from home.

…my concentration is poor and online teaching is tiring, I feel concerned that I have to perform at even higher level to ensure the student get the best from me

R3

I feel l am working harder to prove myself to my employers as I do not want to lose my job. This has resulted in me becoming run down and ill […] I did not take any time off during this time.

R4

Yet I believe I am near to cracking trying to do a full day’s work with the distraction of the virus is really difficult

R14

Furthermore, respondents had an overall negative perception of the change in their eating habits and tended to comment on their consumption of alcohol and sweet foods more than other foods.

I try not to eat from boredom or comfort eat [sic] but I'm not really succeeding. I also drink more alcohol and fizzy drinks, going from almost never to a couple of times a week. I had cut out snacks and drinks like these almost completely in an attempt to lose weight before lockdown, but I feel like the joy of snacking and drinking is more important than losing weight right now.

R7

My appetite is definitely less. I often go without breakfast and have a very small lunch. However, I can binge eat more than before. For example, when I bake, I will eat all that I have made within a day.

R22

Lastly, aspects that were not explored in the survey have emerged as potential barriers to psychological well-being. Some activities that respondents considered beneficial to limit included time spent on news intake and visits to supermarkets. Additionally, lockdown restrictions to exercising were mentioned as problematic.

I have become increasingly anxious when in shops because people are increasingly forgetting to keep their distance

R1

I find news and [sic] media very worrying and negative. I find that sometimes I feel ok and maybe even positive and then I’ll read a bad statistic online or see news headlines and it ruins my mood.

R11

Only being able to exercise once a day was a real issue as it made me feel restricted. My running has reduced due to nervousness about going out and bumping into others as local parks etc have become increasingly busy with other people

R8

I have been confined to my flat, either sitting or lying down most of the times. The restrictions have left me unable to walk as much as I always did before

R10

This theme reveals that the lockdown caused various disruptions to the personal lives of those performing their work duties remotely, including the negative effects of balancing childcare, and their employment fears, which included not performing to “acceptable” standards. Eating habit concerns were also noted, including a report of binging and/or “comfort eating.” Furthermore, challenges affecting mental health were described as key well-being antecedents (and vice versa).

#### Theme 2: Mixed Feelings and Attitudes Toward Remote Working

It was clear that the respondents’ attitudes toward remote working depended on their personal circumstances, and a link between physical health and mental states was observed across narrations. This seemed to also influence what respondents recognized as the challenges or advantages of their remote working dynamic. Physical activity needs, as well as mental health struggles, were reported as challenges. Patterns of more tiredness and lack of sleep during the lockdown were major trends reported among respondents. A lack of ergonomic aids or efficient information technology (IT) resources and the “overuse” of technologies were also reported to negatively impact mental health. Some impracticalities of working from home were reported:

[…] however, everything had to change overnight and that takes time to get right! It has been exhausting, mentally exhausting. I miss the little breaks, walking to a meeting, popping to coffee shop

R5

I am not working as much in the evenings and at weekends. I am behind, though, on my work. In the first couple of weeks of lockdown, I found it difficult to concentrate, adapt, sleep, keep working. I find it hard to mark work online and am fed up of [sic] looking at a computer screen. […] Work online takes about three times as long

R18

The physical difficulties associated with using a dining table desk set-up without proper office equipment (I have an occupational health assessed ergonomic chair at work) have added another layer of challenge

R63

From a personal point of view, I was doing well with exercise but have had some injuries and felt unwell at times. I’ve felt more tired than usual regularly too

R37

Sleep worse than before, cannot switch off at night-time. Have switched from listening to radio 4 to go to sleep to Radio 3 as felt it was constantly information about COVID19

R59

For some, the switch to home working provided limited opportunities for physical activity and blurred the line between work and home life. Several respondents, however, pointed out that a more flexible work dynamic and trust from their employers gave them a greater sense of ownership.

It suits me working more flexibility [sic], my blood pressure is lower, and I have less headaches. […]

R5

the lockdown has not had a negative impact on mental health and has had a positive impact on physical health as we are doing more exercise

R17

Working from home has allowed me to reclaim a few more hours for myself, now that I'm not commuting, and I've been finding ways to make sure that I'm using that time to create a good work/life balance

R19

I am pleased to say that the quality of my life has significantly improved since the COVID outbreak and consequent lockdown. This is because I can work from home and more flexibly, without having to commute and drive/use public transport between cities.

R57

Several respondents further reported some benefits in their physical health and quality of life or work life balance.

In sum, this theme demonstrates that remote working has had both benefits and disadvantages for the work dynamic of the respondents as well as for how they perceive their work-life balance, personal lives, and physical and mental health; all of these factors are key for well-being.

### Theme 3: Aids to Improve Physical and Psychosocial Well-being

This theme focused on various activities and aspects that positively benefited the physical and psychosocial well-being of respondents. Gardening and DIY activities were cited as hobbies that helped respondents to cope with the current stressful situation:

We are still trying to keep active, get fresh air and do DIY at home to balance the body and mind.

R29

Spending more time in the garden which helps to relax, spending more time with pets, learned [sic] new hobbies.

R46

I have tried to keep myself as active as possible, doing work around the house/garden.

R48

Some of the most mentioned benefits of going through this unique circumstance were increasing spirituality, having more contact with nature, self-reflection on life goals, valuing family or a partner’s physical presence, discovering new skills and hobbies, and positive use of the time and resources saved by not commuting:

[…] although it has helped me to focus more on myself [sic] and the things that truly matter

R10

I have still found some elements of lockdown beneficial particularly in the slower pace of life, which has made me think that I may want to keep some aspects of my new routine to improve my mental health when things go back to “normal”

R16

I am grateful for the space in our home, for living with my partner, in the countryside and still being able to go outside. I think I appreciate the “small” things more

R34

I can save money on not having to commute, which helps me because I am the only earner in my household

R57

In summary, this theme encompasses some stressful situations circumvented by the lockdown that were not considered in the closed questions. It additionally identifies coping strategies that several respondents had been employing during the lockdown that had positively influenced their psychosocial well-being.

## Discussion

### Principal Findings

The purpose of the present study was threefold. Firstly, we set out to investigate the relationship between physical and psychosocial well-being and work productivity under lockdown conditions that were imposed as a result of the COVID-19 pandemic. Secondly, we explored whether remote workers with different demographic profiles (eg, gender, parental duties) were differentially affected by lockdown and home-working conditions with respect to their well-being and work productivity. Finally, we aimed to explore remote workers’ perceptions of the lockdown—specifically, its effects on their work productivity and well-being. Key results included (1) the observation of significant relationships between sedentary behavior and poorer mental health, which were in turn related to worse work productivity; (2) exacerbation of these relationships as a consequence of poorer mental health; (3) self-reports of childcare responsibilities (particularly for women), unhealthier diets, work-life balance and home-working environment as barriers to remote working productivity and mental health; (4) self-reports of potential aids and benefits during the lockdown, that researchers, employees, policy makers, etc, can learn from when considering home-working practices. These will now be discussed in turn.

Correlational analyses revealed significant relationships between sedentary behavior (ie, time spent sitting, which in turn was negatively correlated with physical activity, expressed as IPAQ METs) and poorer mental health, which was further related to worse work productivity. To expand, we observed associations between work productivity, mental health, and changes in well-being. For example, we found that higher mental distress scores (K6) were correlated with worse work productivity (IAPT) and worsened well-being (WCI) since the start of the UK lockdown. This is consistent with existing evidence associating work performance and productivity with well-being under nonlockdown conditions [18,19] and demonstrates that the links between physical and mental health observed before the pandemic still explain the variations in these measures and work productivity. It also suggests that recommendations to support remote working that have been proposed in light of past research (eg, adequate IT support, clear communication between staff and management regarding outcomes [18]) still have the potential to be applied in the current situation to improve the productivity of remote workers. Similarly, the current circumstances should prompt broader discussion and policy development concerning the uptake of technology to enable the remote provision of mental health care [63].

Notably, the rates of moderate (55%) and severe (12%) psychological distress were substantially higher in respondents without a mental health diagnosis than has been previously observed in large samples during nonpandemic periods [52] and disasters such as nuclear accidents and earthquakes [64]. Although an element of participant self-selection may explain the extremely high rates of psychological distress we observed, we cannot exclude that the unprecedented magnitude of the ongoing COVID-19 crisis and the prolonged restrictions in many countries, such as the United Kingdom, may be the catalyst for such pronounced reported decreases in psychological well-being.

A further major finding of the current research was that individuals who had received a mental health diagnosis before the lockdown had significantly worse mental health scores, and spent significantly more time sitting, than individuals without a diagnosis. Previous research has identified stress, depression, and anxiety as key predictors of absenteeism (13,800 days lost per annum) in the United Kingdom, resulting in a 6% decrease in productivity [65]. Mental health issues have been reported to affect fundamental aspects of work-life balance [28] and to increase absenteeism and presenteeism [66]. Economic losses as a result of poor mental health have also been documented, further justifying research into cost-effective occupational and psychosocial interventions [67]. Thus, the present findings point to the prevalence of previous and new mental health issues as a crucial consideration of the COVID-19 pandemic, not only for public policy makers when considering management of societal recovery from the pandemic, but also for the private sector to maintain viable working environments. This includes promoting the importance of good well-being and available services that employees can access (without stigma).

Psychological distress and poor mental health, nonetheless, can affect more than just work productivity, and in turn, they can be affected by a variety of environmental stressors. The narrative self-reports revealed that several aspects of respondents’ daily lives during the pandemic (eg, changes to shopping habits and lack of contact with relatives and friends) interacted with other sources of stress or anxiety, which individuals related to poorer mental health. Fear, stress, tiredness, and lack of sleep were widely reported across narrations; and news intake appeared to add to worries and stress. This accords with existing research showing that media and risk-elevating message exposure exacerbated stress, worries, and public anxiety [31,32], but also that news intake correlated with poor mental health in the United Kingdom, particularly at the beginning of the pandemic [30]. Interestingly, however, news intake did not appear to produce significant correlations with mental health (K6) scores or overall well-being change (WCI), although it was correlated with work productivity and was represented as a concern across qualitative comments. As such, other aspects of news consumption not probed in the present survey (eg, how many times per day news is watched; preferred news source or news media type) may be more informative in understanding its effects on mental health, as opposed to simply the number of minutes dedicated to viewing news reports during an average day.

The majority of respondents in our sample (70%) also reported spending a greater amount of time sitting compared to before lockdown restrictions came into effect. Decreasing physical activity for various respondents was partly due to the initial restrictions to outdoor exercise. These findings are important, as even before the COVID-19 pandemic, physical inactivity and sedentary behavior were suggested to be pandemic in their own right, with 31% of individuals aged 15 years or older being identified as physically inactive and approximately 3.2 million deaths per year attributed to these types of behavior [68]. Thus, strategies to circumvent sedation need to be promoted. Encouragingly, however, portions of our sample reported walking more (46%) and engaging in more moderate (45%) and vigorous (52%) exercise.

Similarly, substantial proportions of our sample reported an increase in smoking (63%), alcohol intake (41%), and overall food intake (39%), including sweet treats (53%) and savory snacks (43%); this is consistent with existing research [34] showing more snacking and unhealthy food choices in the general population worldwide during the COVID-19 pandemic. However, we also observed increases in vegetable intake (28%) and home cooking (63%), and a decrease in takeaway use (59%). Our qualitative data suggest that these positive health changes may represent attempts at coping with life and work stressors during the lockdown, a result of more time available, and/or increased awareness of the ill effects of a poor lifestyle, particularly in the context of COVID-19, which have been widely documented during the pandemic [34,38].

Regarding physical activity, our data revealed that some respondents had more time to engage in indoor physical activity than before the lockdown. There is evidence of greater public awareness of the importance of physical activity than ever before [69,70]. Fitness centers have posted free web-based workouts to promote physical activity [43], and information about examples of exercises that can be done at home has been disseminated [71,72]. This includes practical recommendations for aerobic exercise, bodyweight exercises, dance, and active video gaming, as a means to promote physical activity and protect individuals both physically and mentally from COVID-19 [42]. The WHO further highlights how adults and children can achieve the recommended physical activity guidelines at home, with no special equipment and limited space [41]. These recommendations for home-based activities may have been paramount in ensuring that some individuals remained physically active and reduced engagement in sedentary behaviors.

We also explored gender and household characteristics as potential sources of differences in well-being and productivity. Adults living in a household without underage children were significantly more sedentary and, although they engaged in more coping activities, they did not significantly differ on any other metrics as compared to the rest of the sample. More importantly, and consistent with recent research [5], we observed that women were significantly more likely to be the main childcare providers in the household. Although the quantitative analyses did not reveal any significant gender differences in mental health or productivity as a consequence of gender, our qualitative data pointed to childcare duties as a significant challenge for adults—particularly women—who are attempting to maintain their well-being. These childcare responsibilities, which women reported, proved an obstacle to optimal work functioning. However, we did observe that women, compared to men, reported engaging in more recreational activities (eg, cooking/baking, arts and crafts, gardening) in an attempt to preserve their psychological well-being. This could explain why, despite women reporting the challenges of childcare to their psychological health, the quantitative analysis did not reveal differences in well-being as a function of gender. Notably, given the wealth of evidence for existing gender inequalities [9,73-75], research on psychophysical well-being and employment outcomes in remote workers in the aftermath of the pandemic should consider gender an important factor [76]. To circumvent the negative effects of remote working, some of the recreational activities respondents in our sample resorted to (eg, gardening, or meditation) could be further researched as effective strategies to promote good coping/well-being during lockdowns, such as connecting with nature (for a review, see Richardson and colleagues [77]) or embracing a more self-compassionate mindset (for a meta-analysis, see Wilson and colleagues [78]).

Difficulties with maintaining work-life balance were a recurring theme in our qualitative data; however, a more flexible work dynamic and an improved work-life balance were reported in some narrations. Past research [73] has found that voluntary remote working increases work-life balance, observing that remote working can preserve well-being as long as workers can be flexible about it (which is challenging during a lockdown). Mustajab and colleagues [5] further reported a lack of commuting as an advantage of remote working in their sample of Indonesian workers. These findings accord with some of our narrations. However, some of our respondents reported that they were working more hours despite the time saved by not commuting (see also Béland and colleagues [13]). Additionally, and concerningly, respondents in our survey further reported that expectations of productivity levels on the part of their employers were often higher than those required prelockdown. Although flexible employment has previously been found to increase productivity [79], past research did not account for the added stressors of a global pandemic and resulting lockdown (nor autonomy of choice—or lack thereof—to work remotely). An important question leading on from this research is whether the perceived productivity expectation was a requirement of a respondent’s role or a self-assumed expectation. Either way, it has important ramifications regarding employer-employment communications in pandemic and remote-working situations, especially as high-pressure, high-performance work cultures can lead to poorer mental health and staff retention issues [73,80].

Finally, although the International Labour Organization [81] has identified remote working as an excellent strategy to mitigate job losses, and it calls for policies aimed at protecting workers by supplementing their income [82] and encouraging flexible work arrangements [3], many of our respondents reported complications regarding technologies, equipment, and the use of living spaces as a new workplace, which affected their attitudes toward work [83] as well as their ability to work. Such findings are again consistent with existing research [5] indicating a breakdown of communication with managers and colleagues as a common complaint during the COVID-19 pandemic. Thus, adjusting to the new realities of remote work—materially, socially, and psychologically—appears to pose challenges across national economies and cultures.

In summary, currently, there is scant evidence in the literature concerning remote workers’ perceptions of the lockdown’s effects on their physical and psychosocial state and how this might affect their work productivity. This is especially the case for those who were required to transition to remote work during a global pandemic (many of them without being accustomed to this mode of working [83]). However, our qualitative data highlight a range of concerns on the part of respondents, from childcare to perceived work pressures to the practicalities of physically being able to work effectively from home—all of which map onto employment prospects. Notably, employment prospects have been shown to affect mental health, satisfaction, and sense of identity [84], all of which are pillars of psychological well-being [85]. Given the observed relationship between psychological stress and poor work productivity reported by our participants (but also demonstrated via our quantitative data), the current state of affairs for many remote workers could create a negative feedback loop. To expand, the enforced move to remote working, for many, has created work-related uncertainty and pressures, which can negatively affect mental health. The latter, in turn, could then further affect work productivity, exacerbating work-related concerns and, consequently, mental health. Thus, a downward physical, psychological, and work productivity spiral perpetuates.

### Implications

The present study contributes to a nascent field investigating the well-being of remote workers and how remote working can be enhanced. The pandemic recovery process will likely involve a variable period of flexible work arrangements, as some employers may struggle to adapt their workspaces to comply with continued social distancing regulations [86] and some workers might prefer to continue working remotely or via a hybrid office-home model [87]. Importantly, a study [88] conducted among Chinese workers returning to office-based employment following the lifting of restrictions found that ~10% of respondents reported symptoms consistent with a diagnosis of posttraumatic stress disorder. The study found that the incidence of psychiatric symptoms were, among others, the presence of physical symptoms, poor physical health, and a negative perception of a return to the workplace. However, the study also found that the implementation of workplace hygiene and prevention measures (eg, mask-wearing policies) on the part of employers was related to less severe psychiatric symptoms. In light of this, employers, institutional policies, and governments must address the issue affecting workers—both those returning to the workplace, with the perceived vulnerabilities/anxieties this might pose to employees, and those who will continue to work remotely for the foreseeable future. For all modes of working (be it office-based, home-based, or hybrid), all technological and ergonomic aids should be already in place for remote workers to work as closely as possible to their original conditions [83]. Where this is not occurring or cannot be expedited, support structures must be put into place, with employers recognizing that work productivity, rather than increasing, may decrease in the first instance.

Second, childcare responsibilities need greater consideration. Various guidelines have been published to deal with childcare responsibilities [89,90], and calls have been made to support working parents (especially women) in remaining in employment [91]. Current strategies worldwide, however, prioritize changes to individual behaviors without considering the potential impact that employers and working conditions have on worker well-being or the personal circumstances of employees. In light of the relationship between well-being and productivity, it is in the best interest of both workers and employers to consider systemic obstacles to well-being and systemic solutions to them. Expectations of high productivity imposed on workers trying to juggle parental as well as teaching duties while remote working during a time of ongoing or potential school closures can dramatically worsen gender inequalities [91]. Employers should acknowledge the considerable physical and psychological burden on primary child carers (overwhelmingly women) who are balancing remote working with childcare (including home tutoring) responsibilities and implement strategies accordingly.

Dietary recommendations in light of the COVID-19 pandemic [92,93] and particularly for people in lockdown have not been widely formulated and disseminated. Our data revealed increases in overall food intake, specifically the increased consumption of sweet treats and savory snacks and increased frequency of alcohol consumption. However, respondents also reported decreases in takeaway use and increases in home cooking, suggesting there is potential to make remote working a sustainable and healthy lifestyle provided individual and systemic obstacles are investigated and tackled. For example, recent evidence has favored the idea of promoting immunonutrition, rather than only healthy eating, during the current pandemic [94,95]. Although the Better Health campaign in the UK attempts to tackle some systemic barriers, tailoring information that encourages sustainability of a healthy diet across society by guaranteeing access to essential nutrients through healthy eating and/or vitamin supplements is still needed.

Finally, our data point to a clear mental health crisis unfolding in remote workers, which may engender and be engendered by sedentarism and poor nutrition, and in turn may negatively affect work productivity. Public health guidelines for clear and effective actions are needed to improve psychophysical well-being and promote health, thereby also potentially increasing work productivity in the home-working population. There is no shortage of published research to inform such policies in the context of improved nutrition [96,97], exercise [41], mental health [98-100], and work productivity [101,102]. However, evidence-based public health guidelines are only as good as their implementation, which will likely be a function of the material resources both public and private organizations are willing to invest. Future research should continue to promote workers’ physical and psychological well-being, not only as a fundamental goal of public governance but also as a strategic priority for private enterprises and the continued health/wealth of such companies [102].

### Limitations and Future Directions

While the results of our study reveal many findings which could pose important implications for private businesses and public policy, there are important caveats to consider. It should be noted that the survey was distributed via the web. Web-based surveys always include uncertainties about the validity of the data, especially where the survey is self-report and if there are no published studies with a similar or same population to compare to [103]. Nonetheless, web-based surveys have advantages such as decreasing respondents’ inhibitions, offering higher anonymity and increasing the gender, sexual orientation, and diversity of a sample [104].

In attempting to quantify the quality of well-being changes since the start of the lockdown, we could not rely on a standardized, validated measure that probed changes to diet, exercise, and lifestyle. Therefore, we opted to compute an aggregate score (WCI) of distinct questionnaire items on a decrease-increase scale. Despite the lack of formal validation of this scale, the observation of significant correlations between it and standardized measures of productivity (IAPT) and psychological distress (K6) is indicative of both construct and criterion validity. Future work should explore and improve the psychometric properties of this instrument.

In terms of statistical power, our study had a sufficient sample size to detect correlations of *τ*_b_>0.3 with 0.8 power at an *α* of .005 but may have had less power to detect true effects for our smaller correlations at the same *α* level. We nevertheless opted to adopt this more stringent *α* level given the number of correlational tests we conducted. While even the smaller correlations we observed were interpretable in light of the existing literature (and additional correlations were significant at lower *α* levels), future research should aim for larger samples to achieve greater statistical power and to possibly enable the analysis of individual differences. Indeed, in recruiting larger samples, future studies should seek to differentiate the type of remote worker occupation enabling fuller analysis of the particular struggles of different worker groups [8]. In addition, adding focus group or semistructured interview methods would add to the robustness, richness, and depth of any findings [105,106], especially concerning a novel topic such as this. Indeed, to our knowledge, this is the first study that considers a comprehensive overview of well-being and its effects on remote-working productivity in a UK population.

### Conclusion

The mass switch to working remotely during COVID-19 lockdown, and the many worries stemming from the pandemic, have been argued to adversely affect the physical and mental well-being of workforces globally. The results of the current study demonstrate that well-being, which has a significant impact on productivity, is at stake when it comes to working remotely during a pandemic. The main findings of the current study were a relationship between sedentary behavior and poorer mental health, with negative effects on work productivity; moreover, challenges to productive remote working ranging from IT provisions to parental obligations were observed. Therefore, policies that promote physical activity, reduce psychological distress, address gender gaps, and support balancing childcare/home schooling while working remotely are urgent. It is also essential that employers monitor workers’ well-being and implement systemic guidelines and practices to maintain worker well-being (eg, encouraging physically active breaks, providing more logistic support) while also promoting individual lifestyle changes (eg, meditation, healthy cooking), as well as policy related to reasonable adjustments in the “new” workplace and clear productivity expectations. Targeted strategies such as these to support people working remotely as a consequence of COVID-19 may help to thwart, or at least attenuate, an international public health crisis. To this end, findings from well-being research also need to be made easily accessible to remote workers and companies.

## Abbreviations

IAPT: Brief Instrument to Assess Workers’ Productivity During a Working Day
IPAQ: International Physical Activity Questionnaire
IT: information technology
K6: Kessler-6 Distress Scale
MET: metabolic equivalent
WCI: well-being change index
WHO: World Health Organization
